# Examination of Dysglycaemia among Newly Diagnosed Tuberculosis Patients in Ghana: A Cross-Sectional Study

**DOI:** 10.1155/2018/1830372

**Published:** 2018-09-24

**Authors:** Ernest Yorke, Vincent Boima, Ida Dzifa Dey, Yacoba Atiase, Josephine Akpalu, Alfred Edwin Yawson, Vincent Ganu, Audrey Forson, C. Charles Mate-Kole

**Affiliations:** ^1^Department of Medicine & Therapeutics, School of Medicine & Dentistry, College of Health Sciences, University of Ghana, Ghana; ^2^Department of Community Health, School of Public Health, University of Ghana, Ghana; ^3^Department of Medicine, Korle-Bu Teaching Hospital, Accra, Ghana; ^4^Department of Psychiatry, School of Medicine & Dentistry, College of Health Sciences, Korle-Bu, Ghana; ^5^Centre for Ageing Studies, College of Humanities, University of Ghana, Ghana; ^6^Department of Psychology, College of Humanities, University of Ghana, Ghana

## Abstract

The burden of both tuberculosis (TB) and diabetes mellitus in developing countries including Ghana is high; often, the two coexist and impact each other negatively.* Objective*. The study aimed to determine the prevalence and predictive factors of dysglycaemia among newly diagnosed smear positive tuberculosis patients at a tertiary tuberculosis treatment centre in Ghana.* Methods*. Dysglycaemia at diagnosis was determined by the use of oral glucose tolerance test (OGTT), while sputum smear microscopy was used to assess the sputum status. Only smear positive patients were included in the study. Information on sociodemographic, anthropometrical, clinical, and medication history was also obtained.* Results*. In all, 146 participants, aged 18 to 75 years with a mean age of 38.7 years comprising 115 (78.8%) males and 31 (21.2%) females, were involved in the analysis. Upon initial screening, using fasting plasma glucose (FPG), 91.1 % had normal fasting level, 5.5 % had impaired fasting, and 3.4% were diagnosed with diabetes. Using 2-hour postprandial values (2HPP), 59.6% had normal plasma glucose, 28.8 % had impaired glucose tolerance (IGT), and 11.6 % were diagnosed with diabetes. Overall, the prevalence of dysglycaemia (i.e., impaired fasting and diabetes) was 8.9% (95% CI: 5.21–14.82%) with FPG test and 40.4% (95% CI: 32.68–48.65%) with 2HPP test. The analysis revealed that 2HPP was associated with high mean age compared to FPG (36.67 ± 13.97 versus 41.69 ± 13.97, p-value = 0.033). In addition, marital status was significantly associated with FPG status of patients (p = 0.028).* Conclusion*. The prevalence of dysglycaemia was high among smear positive TB patients in Ghana. Higher mean age and marital status were associated with abnormal glucose tolerance and fasting plasma glucose, respectively. Clinical management of patients with tuberculosis should include screening for diabetes.

## 1. Introduction

Tuberculosis (TB) infections continue to be a concern worldwide and it remains a deadly communicable disease. The World Health Organisation (WHO) estimated that 10.4 million new cases of TB occurred and 1.4 million died from the disease in 2015 despite several preventive strategies to reduce the burden and impact [1]. Over 70% of these new cases occurred in developing countries with the African region experiencing the highest rate of death relative to the population [1].

Diabetes and TB as separate disease entities impact negatively on each other [2]. Although at the population level, HIV is the most important risk factor for TB [1], diabetes, which causes impaired immunity, has long been recognized as a risk factor for infections including TB [3]. Diabetes is a risk factor for lower respiratory infections including TB. A review by Steven et al. reported that diabetes increases TB risk by 1.5- to 7.8-fold [4]. Another meta-analysis by Joen et al. reported that the relative risk for TB among diabetes patients was 3.11 [5]. They stated that the prevalence of diabetes ranged from 1.9% to 35% after screening TB patients; the highest rates were among regions of the world with the highest diabetes prevalence [5]. Also, a study from United States of America reported that the odds ratio of multidrug resistant (MDR) TB associated with diabetes patients is 2.1 [6].

Diabetes may affect the presentation of TB by being associated with more cavitations [6], lower lobe involvements [2], higher rates of haemoptysis [6], fever [6], increased risk of treatment failure, relapse, and death[7]. Further, diabetes may affect the pharmacokinetics of rifampicin, a major component of anti-TB medications, and reduce its plasma concentration [8]. However, it is not clear whether the reduced plasma concentrations of rifampicin affect treatment outcomes [2, 8].

Studies have reported that TB could lead to impaired glucose tolerance (IGT) [9] and new onset diabetes [10, 11]. Generally, IGT normalises after the TB has been successfully treated, but it remains a significant risk factor for developing type 2 diabetes in the future [12].

This hyperglycaemia caused by TB infection may lead to overdiagnosis of diabetes in previously unscreened TB patients [3] and can also worsen glycaemic control in previously diagnosed diabetes patients [12]. The latter situation may, therefore, warrant adjustment in doses of antiglycemic agents or a complete switch to insulin therapy.

The present study examined the burden of dysglycaemia among newly diagnosed smear positive tuberculosis patients at a tertiary tuberculosis treatment centre and explored factors that might predict dysglycaemia among these patients.

## 2. Materials and Methods

### 2.1. Study Design and Site

The study was a hospital based cross-sectional study carried out at the Chest Clinic of the Korle-Bu Teaching Hospital, a tertiary care facility and the largest hospital in Ghana. The Korle-Bu Chest Clinic serves as the main referral centre for respiratory and TB cases for the southern part of the country.

### 2.2. Participants

Participants were newly diagnosed smear positive TB patients that were referred to the treatment centre of Korle-Bu Chest Clinic.

Patients aged 18 years and over with no previous history of TB treatment were recruited. All the participants gave informed consent. Exclusion criteria indicated participants with smear negative TB cases, those with extrapulmonary TB or those who refused consent.

### 2.3. Sampling

All smear positive TB patients who met the inclusion criteria were recruited on weekdays over a 12-month period. One hundred and ninety smear positive TB patients were sampled at the end of the study period, of which 44 did not stay to complete the full OGTT test or refused consent.

### 2.4. Measurements

At study enrolment, participants were administered a questionnaire to assess and document demographic and anthropometric characteristics as well as medical history

Calculated body mass index (BMI) was categorized as obesity, overweight, normal, and underweight and were defined as 30.0 or more, 25-29.9, 18.5-24.9, and less than 18.5 (Kg/m^2^), respectively [13].

A 75 g oral glucose tolerance test (OGTT) was used to determine impaired glucose tolerance (IGT), impaired fasting glucose (IFG), and diabetes at diagnosis. 10ml fasting blood sample was taken into fluoridated blood sample tubes (kept on ice and centrifuged within 15 minutes of blood draw), ethylene-diamine-tetra-acetic acid (EDTA) tubes, and plain tubes [14]. Patients then received 75g glucose in 250ml water, and after 2 hours blood sample was taken into fluoridated sample tubes and processed similarly as the fasting sample. Plasma glucose was determined by glucose oxidase commercial reagent kits and controls (DiaSys, GmbH, Germany). Diagnoses of impaired fasting and glucose tolerance were made when FPG and 2HPP values are 6.1-6.9 mmol/l and 7.8-11 mmol/l, respectively. Diabetes was diagnosed when FPG and 2HPP are >7mmol/l and >11.1 mmol/l, respectively, or on regular medication for diabetes. FPG and 2HPP values below 6.1 mmol/l and 7.8 mmol/l are normal [15].

Microscopy for acid fact bacilli (AFBs) was performed using Ziehl-Neelsen (ZN) stain. Patients found to have diabetes were referred to receive appropriate care.

### 2.5. Ethical Considerations

All patients provided written informed consent. The study received approval from the University of Ghana College of Health Sciences Ethics and Protocol Review Committee prior to the commencement of the study with reference number URF/9/ILG-076/2015-2016.

We complied with the Helsinki Declaration of 1964 (Revised 2013) on human experimentation. Strict confidentiality of data and privacy for study participants were ensured. Data was kept secured and was available only to the principal investigator.

### 2.6. Statistical Analysis

Data was entered into Microsoft Excel 2010 version and imported into the Statistical software STATA version 15 for analysis. Analysis was done for 146 patients with complete data.

Descriptive statistics of the sociodemographic, anthropometric, clinical and glycaemic variables were summarised as frequencies, percentages, means, and standard deviations.

Chi-square and Fisher's exact tests were used to test for association between the plasma glucose status of participants (categorized as normal and abnormal plasma glucose level) and other categorical independent variables; while t-test was used for comparing means of continuous variables among smear positive TB patients with normal and abnormal plasma glucose levels.

Binary logistic regression models and Poisson regression model were used to test the effect of sociodemographic, clinical, and lifestyle factors on the outcome variables, 2HPP and FBG, respectively. Poisson model was chosen over logistic regression model because of the very low prevalence of abnormal FBG glucose level [16]. Potential for effect modification was examined by testing the significance of interactions. Only statistically significant interaction terms were retained in the final models. The statistical tests were set at 5% significance level.

## 3. Results

One hundred and forty-six (146) participants, aged 18 to 75 years with a mean age of 38.7 years comprising 115 (78.8%) males and 31 (21.2%) females, were involved in the analysis. Seven out of every ten selected smear positive TB patients were underweight. Mean values (± SD) of some clinical parameters such as weight, height, waist circumference, hip circumference, systolic blood pressure, and diastolic blood pressure are 52.74 ± 8.94 Kg, 1.68 ± 0.08 m, 76.25 ± 7.32 cm, 89.37 ± 7.43 cm, 117.61 ± 21.76 mmHg, and 77.13 ± 12.10 mmHg, respectively. Detailed distribution of sociodemographic, anthropometric, and other clinical characteristics of the participants can be found in Table 1.

### 3.1. Prevalence of Diabetes and Dysglycaemia

After screening, using fasting plasma glucose, 91.1 % had normal fasting level, 5.5 % had impaired fasting, and 3.4% were diagnosed to be diabetic. Using 2-hour postprandial values (2HPP), 59.6% had normal plasma glucose, 28.8 % had impaired glucose tolerance, and 17 out of 146 (11.6 %) had diabetes. In all, the combined prevalence of dysglycaemia using fasting values was 8.9% (95% CI: 5.21–14.82%) (i.e., impaired fasting and diabetes) while that for postprandial values (impaired glucose tolerance and diabetes) was 40.4% (95% CI: 32.68–48.65%) (Table 1).

### 3.2. Association between Sociodemographic Factors, Clinical Factors, Lifestyle Factors and 2HPP Status

The bivariate analysis of factors associated with 2HPP status (Table 2) revealed that mean age was significantly associated with 2HPP status, where those with abnormal 2HPP had higher mean age (36.67 ± 13.97  vs  41.69 ± 13.97, p − value = 0.033).  

### 3.3. Association between Sociodemographic Factors, Clinical Factors, Lifestyle Factors and FPG Status

The bivariate analysis of factors associated with FPG status (Table 3) revealed that marital status was associated with FPG status of patients; the proportion of patients with abnormal FPG was significantly higher among separated/divorced participants (33.3%), while the married ones had the least 6.5% (*χ*^2^ = 7.16, p = 0.028). The other factors did not have any statistically significant association with FPG status (p>0.05). See Table 3.

### 3.4. Evaluating Effects of Sociodemographic Factors on 2HPP Status

The results from multiple binary logistic regression analysis showed that none of the sociodemographic factors was statistically significant in predicting the 2HPP status of patients. Refer to Table 4. The effects of sociodemographic factors on 2HPP status were assessed in six different nested models with only sociodemographic characteristics of the study participants. These models were assessed and evaluated with Area under Receiver Operating Characteristic Curve (AUROC) and Akaike Information Criterion (AIC).

Although, statistically, AUROC did not vary significantly across the six nested models (*χ*^2^ = 5.07, p = 0.408), model 6 was the best performing model (AUROC=69.9%, AIC=208.6). Detailed evaluation of the six models and graph showing the performance of the models can be found under* Supplementary Materials *I* & *II, respectively.

Analysis investigated the effect of clinical, lifestyle, and socioeconomic factors on 2HPP status. After controlling for sociodemographic factors, there is no enough statistical evidence to conclude that any of the clinical and lifestyle factors have significant effect on 2HPP status (p>0.05) (Table 5). The detailed assessment of the statistical models used based on Area under Receiver Operating Characteristic Curve (AUROC) and Akaike Information Criterion (AIC) is shown under* Supplementary Materials *III.

### 3.5. Effects of Sociodemographic, Clinical, and Lifestyle Factors on FPG Status

The results from the Poisson regression model showed that there is no enough statistical evidence to conclude that any of the sociodemographic factors, clinical conditions, and lifestyle factors have significant effect on FPG status of patients (p>0.05), Table 6.

## 4. Discussion

We found a high prevalence of dysglycaemia in our study (i.e., 8.9% and 40.4 %) by fasting and postprandial values, respectively, as well as diabetes of 3.4% and 11.6 % by fasting and 2-hour post-glucose values, respectively. These findings were similar to other studies where 5-30 % of TB patients were found to have diabetes [17–19].

Diabetes is significant risk factor for TB at the population level [3] and increases TB risk by 1.5 to 7.8 times [4]. A study by Joen et al. found that the relative risk for TB among diabetes patients was 3.11 [5], while a review by Alisjahbana et al. [6] suggested that the odds ratio of multidrug resistant (MDR) TB associated with diabetes patients is 2.1. Joen et al. [5] in a subsequent study reported that after screening TB patients, diabetes prevalence ranged from 1.9% to as high as 35%. They observed that rates were higher in regions of the world where diabetes prevalence was also higher.

Diabetes impact on TB as a disease entity and its management in many ways. Some studies have shown that diabetes patients tend to show more lower lobe involvement than their nondiabetic counterparts due to reactivation of old foci [2]. Alisjahbana et al. reported lower rates of cavitations in some studies while others have higher rates [6]. Comorbid TB and diabetes may also be associated with higher rates of haemoptysis, fever, and atypical presentations [6]. Baker et al. in a systematic review reported that patients with diabetes have a risk ratio (RR) for the combined outcome of failure and death of 1.69 (95% CI, 1.36 to 2.12), while the RR of death during tuberculosis treatment among 23 unadjusted studies was 1.89 (95% CI, 1.52 to 2.36) [7]. In this same review, diabetes was also associated with an increased risk of relapse (RR, 3.89; 95% CI, 2.43 to 6.23) [7].

Diabetes can affect the pharmacokinetics of anti-TB drugs especially rifampicin and reduce their plasma concentrations [8]. There are however conflicting reports regarding whether the efficacy of TB treatment is affected by this interaction [2, 8]. Consequently, the regimen for treatment of TB among both diabetics and nondiabetics is the same [3, 8]. Also, the general approach to management of diabetes does not differ in the presence of TB or not. The treatment of diabetes with concomitant TB infection requires careful evaluation and choice of antiglycemic medication [1, 3]. In many situations, insulin is the preferred agent in type 2 diabetes where there is active TB infection [3]. Appropriate diet advice is also needed, aimed at balancing glycaemic control and the nutritional demands of largely underweight and malnourished TB patients [20].

Patients with abnormal 2HPP were more likely to have higher mean age values in this study. This may be a reflection of an underlying increased risk of type 2 diabetes which is prevalent among older people [21]. The incidence of diabetes increases with age until about the age of 65 years, after which both incidence and prevalence tend to plateau [21]. This group of patients tend to have multiple comorbidities including diabetic microvascular and macrovascular complications, coupled with reduced functional status, and increased risk of institutionalization [21, 22]. They also tend to be the productive work force and major family income earners. Additionally, older adults are more likely to develop extra-pulmonary and atypical disease patterns that are often harder to diagnose than conventional sputum smear positive pulmonary tuberculosis [23]. Consequently such patients with TB and diabetes are more likely to suffer worse morbidity, mortality, and social impact [1, 21, 23].

Marital status was significantly associated with FPG status of patients; the proportion of patients with abnormal FPG turns to be high among patients who are separated/divorced (33.3%), while the married ones had the least (6.5%). It may be possible that stress among smear positive TB patients who are separated/divorced predisposes them to a higher risk of dysglycaemia. It is noteworthy that the numbers in the separated/divorced group were small and may not reflect the true situation.

The findings of most participants in this study, being male, young age, and single, are well known characteristics of TB patients [1, 24, 25]. Other risk factors include low-income earners, bacillary load, proximity to a person with active TB, malnutrition, alcohol abuse, smoking, overcrowding, HIV infection, diabetes, and immunosuppressive drug [1, 24, 25]. The participants also had low average BMI of 18.49 ± 3.00 Kg/m^2^ with about 70 % being underweight. TB patients tend to come from low socioeconomic background and generally malnourished and underweight [26]. The inflammatory response to infection [27, 28] as well as nausea, loss of appetite, and vomiting associated with TB is possible contributor to weight loss. Theoretically, diabetes may compound weight loss. Diabetes is associated with reduced insulin secretion or action, which results in an increased catabolic state with breakdown of body tissue and loss of calories leading to weight loss. However, in our study, there were no statistical differences in the mean BMI among those with normal and abnormal glucose values (both FBS and 2HPP).

## 5. Conclusion

The study found a high prevalence of dysglycaemia among smear positive TB patients similar to many previous studies on the subject. Higher mean age and marital status were associated with abnormal glucose tolerance and fasting plasma glucose, respectively. It is one of the very few studies done on this subject in the African subregion.

The findings of the study come to support the increasing recognition of the association between TB and diabetes. With a high burden of TB and increasing prevalence of diabetes in Africa, the findings also reinforce the call for the institutionalization of screening for diabetes among newly diagnosed TB patients in this part of the world.

## 6. Limitations

This study only described dysglycaemia at diagnosis and associated factors. Follow-up studies are needed to ascertain treatment outcomes among TB patients with dysglycaemia and those without.

## Figures and Tables

**Table 1:** Background characteristics of study participants.

	**Frequency**	**Percentage**
Age, years (mean ± SD)	38.70 ± 13.97	
**Sex**		
Male	115	78.80
Female	31	21.20
**Marital Status**		
Married	62	42.47
Single	75	51.37
Separated/Divorced	9	6.16
**Highest educational level**		
None	13	8.90
Primary	14	9.59
Middle School/ JHS	54	36.99
O-Level/A-level/SHS	32	21.92
Tertiary	23	15.75
Other	10	6.85
**Employment status**		
Unemployed	38	26.03
Employed	108	73.97
**Lifestyle factors **		
**Smoking**		
Yes	10	6.85
No	136	93.15
**Alcohol intake**		
Yes	22	15.07
No	124	84.93
**Clinical factors**		
**BMI (Kg/m** ^**2**^ **)**		
Mean ± SD	18.49 ± 3.00	
Below 18.5 (Underweight)	103	70.55
18.5-24.9 (Normal)	40	27.4
25-29.9 (Over Weight)	2	1.37
Above 30 (Obese)	1	0.68
**Glycaemic Variables**		
**Fasting Plasma Glucose (mmol/L)**		
(mean ± SD)	5.12 ± 1.52	
Normal (<6.1)	133	91.1
Impaired (6.1-7)	8	5.48
Diabetes (>7.1)	5	3.42
**2 HPP Glucose (mmol/L)**		
(mean ± SD)	8.35 ± 3.44	
Normal (<6.1)	87	59.59
Impaired (6.1-7)	42	28.77
Diabetes (>7.1)	17	11.64

**Table 2:** Association between sociodemographic factors, clinical factors, and lifestyle factors and 2HPP Status.

	2HPP			
	**Normal**	**Abnormal**	**χ** ^2^	**p-value**
Age **(Mean ± SD)**	36.67 ± 13.97	41.69 ± 13.97	-2.15 (t-test)	0.033*∗*
Sex			1.09	0.297
**Male**	66 (57.39)	49 (42.61)		
**Female**	21 (67.74)	10 (32.26)		
Marital Status			1.21	0.546
**Married**	36 (58.06)	26 (41.94)		
**Single**	47(62.67)	28 (37.33)		
**Separated/Divorced**	4 (44.44)	5 (55.6)		
Highest Educational Level			9.65	0.086
**None**	9 (69.23)	4 (30.77)		
**Primary**	8 (57.14)	6 (42.86)		
**Middle School/ JHS**	24 (44.44)	30 (55.56)		
**SHS/ O-level**	23 (71.88)	9 (28.13)		
**Tertiary**	17 (73.91)	6 (26.09)		
**Other**	6 (60.00)	4 (40.00)		
Employment Status			0.82	0.365
**Unemployed**	25 (65.79)	13 (34.21)		
**Employed**	62 (57.41)	46 (42.59)		
Lifestyle Factors				
Smoking				0.74 §
**Yes**	7 (70.00)	3 (30.00)		
**No**	80 (58.82)	56 (41.18)		
Alcohol Intake			0.18	0.675
**Yes**	14 (63.64)	8 (36.36)		
**No**	73 (58.87)	51(41.13)		
Clinical Factors				
**Weight (Mean ± SD), Kg**	53.72 ± 8.53	51.31 ± 9.41	1.58	0.117
**Height (Mean ± SD), m**	1.67 ± 0.08	1.68 ± 0.08	0.85	0.40
BMI (Kg/m^2^)				
**Mean ± SD**	18.80 ± 2.73	18.02 ± 3.33	1.51	0.135
**Below 18.5 (Underweight)**	59 (57.28)	44 (42.72)		0.369
**18.5-24.9 (Normal)**	26 (65.00)	14 (35.00)		
**25-29.9 (Over Weight)**	2 (100.00)	0		
**Above 30 Obese)**	0	1(100.00)		
Waist and Hip Circumference, cm	76.00 ± 7.10	76.62 ± 7.68	-0.5	0.619
**Waist Circumference (Mean ± SD)**				
**Hip Circumference (Mean ± SD)**	89.53 ± 7.65	89.14 ± 7.13	0.31	0.756
**Waist-Hip Ratio (Mean ± SD)**	0.86 ± 0.06	0.86 ± 0.08	-0.22	0.824
Blood Pressure (mmHg)	116.39 ± 17.43	119.42± 26.99	-0.76	0.449
**SBP (Mean ± SD)**				
**DBP (Mean ± SD)**	76.76 ± 11.99	77.66 ± 12.35	-0.44	0.664
**Pulse (Mean ± SD)**	103.10 ± 15.81	107.10± 18.40	-1.36	0.176

*∗*p<0.05; values were based on Pearson chi-square and Fisher's exact test for categorical variables, and t-test for comparing the means. SD: standard deviation of age. (%) represents row percentage and § p-value estimate from Fisher's exact test.

**Table 3:** Association between sociodemographic factors, clinical factors, and lifestyle factors and FPG Status.

	FBG			
	**Normal**	**Abnormal**	**χ** ^2^	**p-value**
**Age** (Mean ± SD)	38.29 ± 13.84	42.85 ± 15.21	-1.04 (t-test)	0.316 *ǂ*
**Sex**				1.00 §
Male	105(91.30)	10(8.70)		
Female	28(90.32)	3(9.68)		
**Marital Status**			7.16	0.028*∗*
Married	58 (93.55)	4 (6.45)		
Single	69 (92.00)	6 (8.00)		
Separated/Divorced	6 (66.67)	3 (33.33)		
**Highest Educational Level**				0.433 §
None	12(92.31)	1 (7.69)		
Primary	13(92.86)	1 (7.14)		
Middle School/ JHS	47(87.04)	7 (12.96)		
O-level/SHS	31(96.88)	1 (3.13)		
Tertiary	22(95.65)	1 (4.35)		
Other	8(80.00)	2 (20.00)		
**Employment Status**				0.185 §
Unemployed	37(97.37)	1 (2.63)		
Employed	96(88.89)	12(11.11)		
**Smoking**				1.00 §
Yes	9(90.00)	1(10.00)		
No	124(91.18)	12(8.82)		
**Alcohol Intake**				0.692 §
Yes	21(95.45)	1 (4.55)		
No	112(90.32)	12(9.68)		
**Clinical Factors**	53.05 ± 9.02	49.62 ± 7.75	1.5	0.153 *ǂ*
Weight (Mean ± SD)				
Height (Mean ± SD)	1.69 ± 0.08	1.66 ± 0.06	1.44	0.168 *ǂ*
**BMI**				
Mean ± SD	18.52 ± 3.02	18.12 ± 2.92	0.47	0.647 *ǂ*
Below 18.5 (Underweight)	94 (91.26)	9 (8.74)		0.816 §
18.5-24.9 (Normal)	36 (90.00)	4 (10.00)		
25-29.9 (Over Weight)	2 (100.0)	0		
Above 30 (Obese)	1 (100.0)	0		
**Waist Circumference**				
Waist Circumference (Mean ± SD)	76.30 ± 7.48	76.21 ± 5.72	0.05	0.963 *ǂ*
Hip Circumference (Mean ± SD)	89.55 ± 7.48	88.06 ± 6.87	0.74	0.472 *ǂ*
Waist-Hip Ratio (Mean ± SD)	0.85 ± 0.07	0.86 ± 0.07	-0.3	0.769 *ǂ*
**Blood Pressure**				
SBP (Mean ± SD)	117.36 ± 21.17	120.23± 28.05	-0.36	0.725 *ǂ*
DBP (Mean ± SD)	76.80 ± 12.08	80.5 ± 12.35	-1.04	0.318 *ǂ*
**Pulse** (Mean ± SD)	104.15 ± 17.17	110.54 ± 13.93	-1.54	0.142 *ǂ*

*∗*p<0.05; values were based on Pearson chi-square and Fisher's exact test for categorical variables, and t-test for comparing the means. SD: standard deviation of age. (%) represents row percentage, *ǂ* estimated p-value from the Welch t-test, and § p-value estimate from Fisher's exact test.

**Table 4:** Effects of sociodemographic factors on 2HPP status (model six).

	**OR**	**95**%** IC**	**p-value**
Current age	1.03	0.99 - 1.06	0.118
**Highest educational level**			0.196
None	Ref		
Primary	1.58	0.3 - 8.36	
Middle School/ JHS	2.32	0.56 - 9.62	
O-Level/SHS	0.75	0.16 - 3.59	
Tertiary	0.71	0.14 - 3.64	
Other	1.04	0.15 - 7.12	
**Employment status**			
Unemployed	Ref		
Employed	1.60	0.66 - 3.92	0.300
**Sex**			
Male	Ref		
Female	0.69	0.27 - 1.73	0.427
**Marital status**			0.790
Married	Ref		
Single	1.32	0.54 - 3.22	
Separated/Divorced	1.37	0.3 - 6.29	

**Table 5:** Evaluating the effect of clinical and lifestyle factors on 2HPP.

	**2HPP**			**2HPP**		
	**Unadjusted effect of clinical and lifestyle factors on 2HPP: model 11**			Effect of clinical and lifestyle factors on 2HPP controlling for Sociodemographic factors: model 12		
	**OR**	**95**%** CI**	**p-value**	**aOR**	**95**%** CI**	**p-value**
BMI	0.82	0.68 - 0.97	0.024	0.86	0.7 - 1.05	0.141
Waist circumference	1.05	0.98 - 1.14	0.168	1.02	0.94 - 1.12	0.627
Hip circumference	1.00	0.92 - 1.09	0.949	1.00	0.91 - 1.09	0.937
Systolic BP	1.01	0.98 - 1.04	0.497	1.00	0.97 - 1.03	0.819
Diastolic BP	1.00	0.95 - 1.05	0.997	1.01	0.96 - 1.06	0.783
Smoking						
No	ref					
Yes	0.52	0.1 - 2.68	0.433	0.35	0.05 - 2.36	0.284
Alcohol intake						
No	ref					
Yes	0.90	0.29 - 2.83	0.862	0.69	0.18 - 2.58	0.579

**AUROC (95**%** CI)**	63.16% (53.88 - 72.43)			73.39% (65.29 - 81.48)		
**AIC**		204.7			216.85	
**HL GOF**	*χ* ^2^= 4.96	p=0.762		*χ* ^2^= 7.01	p=0.536	

OR: odds ratio, aOR: adjusted odds ratio, CI: confidence interval, Ref: reference category, *∗*p<0.05, *∗∗*p<0.01, *∗∗∗*p<0.001, AUROC: Area under receiver operating characteristic curve, AIC: Akaike Information Criterion, HL GOF: Hosmer Lemeshow Goodness of fit test.

**Table 6:** Effects of sociodemographics factors, clinical conditions, and lifestyle factors on FPG status.

	**Unadjusted**			**Adjusted**		
	**PR**	**95% CI**	**p-value**	**PR**	**95% CI**	**p-value**
Current age	1.02	0.98 - 1.06	0.287	1.00	0.94 - 1.06	0.958
**Highest educational level**		-	0.603		-	0.494
None	Ref					
Primary	0.93	0.06 - 14.85		0.67	0.03 - 14.83	
Middle school/JHS	1.69	0.21 - 13.7		2.40	0.23 - 25.2	
SSS/O-level/SHS	0.41	0.03 - 6.49		0.33	0.02 - 7.02	
Tertiary	0.57	0.04 - 9.04		0.66	0.04 - 12.03	
Other	2.60	0.24 - 28.67		3.00	0.18 - 50.36	
**Employment status**		-	0.166		-	
Unemployed	Ref					
Employed	4.22	0.55 - 32.47		7.73	0.87 - 68.57	0.066
**Sex**		-	0.871		-	
Male	Ref					
Female	1.11	0.31 - 4.04		0.95	0.2 - 4.6	0.953
**Marital status**		-	0.067		-	0.165
Married	Ref					
Single	1.24	0.35 - 4.39		1.12	0.21 - 5.92	
Separated/Divorced	5.17	1.16 - 23.08		6.12	0.9 - 41.56	
**BMI**	0.96	0.79 - 1.16	0.661	0.96	0.68 - 1.35	0.803
Waist Circumference	1.00	0.93 - 1.08	0.999	1.05	0.92 - 1.2	0.460
Hip Circumference	0.98	0.91 - 1.05	0.534	0.95	0.82 - 1.1	0.522
**Systolic BP**	1.00	0.98 - 1.03	0.663	0.96	0.91 - 1.02	0.217
Diastolic BP	1.02	0.98 - 1.06	0.314	1.06	0.96 - 1.16	0.245
**Smoking**		-	0.904		-	0.427
No	Ref					
Yes	0.88	0.11 - 6.79		3.94	0.13 - 115.79	
**Alcohol intake**		-	0.468		-	0.289
No						
Yes	2.13	0.28 - 16.37		0.17	0.01 - 4.44	

Ref: the reference category, PR: prevalence ratio from the multiple Poisson regression model, CI: confidence interval. p<0.05, *∗∗*p<0.01, *∗∗∗*p<0.001.

## Data Availability

The data used to support the findings of this study are available from the corresponding author upon request.

## Abbreviations

AFB: Acid fact bacilli
AIC: Akaike Information Criterion
aOR: Adjusted odds ratio
AUROC: Area under Receiver Operating Characteristic Curve (AUROC)
BP: Blood pressure
BMI: Body mass index
CI: Confidence Interval
EDTA: Ethylene-diamine-tetra-acetic acid
FPG: Fasting plasma glucose
HIV: Human Immunodeficiency virus
HL GOF: Hosmer Lemeshow Goodness of fit test
IGT: Impaired glucose tolerance
MDR: Multidrug resistant tuberculosis
OGTT: Oral glucose tolerance test
OR: Odds ratio
SD: Standard deviation
TB: Tuberculosis
WHO: World Health Organisation
ZN: Ziehl-Neelsen
2HPP: 2-hour postprandial glucose.

## Supplementary Materials

Supplementary Materials I, II: comparison of the performance of six nested models used in assessing the effect of sociodemographic factors on 2HPP (2-hour postprandial glucose) status. III: comparison of the performance of two nested models used in assessing the effect of clinical and lifestyle factors on 2HPP (2-hour postprandial glucose) status.
