# Supplementary material for: Examination of Dysglycaemia among Newly Diagnosed Tuberculosis Patients in Ghana: A Cross-Sectional Study
Source: Tuberc Res Treat. 2018 Sep 24;2018:1830372. doi: 10.1155/2018/1830372 (PMC6174760; doi:10.1155/2018/1830372)

SUPPLEMENTARY MATERIAL I

Detailed evaluation of the effects of socio-demographic factors on 2HPP status of the study participants using six nested models.

	2HPP Status					
	**Model 1**	**Model 2**	**Model 3**	**Model 4**	**Model 5**	**Model 6**
Predictors	Age	Age	Age	Age	Age	Age
		Educational level	Educational level	Educational level	Educational level	Educational level
			Employment status	Employment status	Employment status	Employment status
				Gender	Gender	Gender
						Marital status
**Model performance index**						
AUROC (95%CI)	60.14% (50.66 - 69.62)	67.19% (58.36 – 76.03)	68.00% (59.31 – 76.70)	68.02% (59.32 – 76.73)	69.13% (60.65 – 77.61)	69.88% (61.50 – 78.26)
AIC	196.40	198.85	198.85	200.30	205.06	208.58
HL GOF	$\chi^2 = 5.70$ $p = 0.681$	$\chi^2 = 8.68,$ $p = 0.370$	$\chi^2 = 4.15,$ $p = 0.843$	$\chi^2 = 5.25,$ $p = 0.731$	$\chi^2 = 12.68,$ $p = 0.123$	$\chi^2 = 12.47,$ $p = 0.132$

2HPP: 2-hour postprandial glucose, *AUROC: Area under receiver operating characteristic curve, AIC: Akaike Information criterion, HL GOF: Hosmer Lameshow Goodness of fit test.*

SUPPLEMENTARY MATERIAL II

Comparing the performance of six nested models used in assessing the effect of socio-demographic factors on 2HPP (*2-hour postprandial glucose*) status.

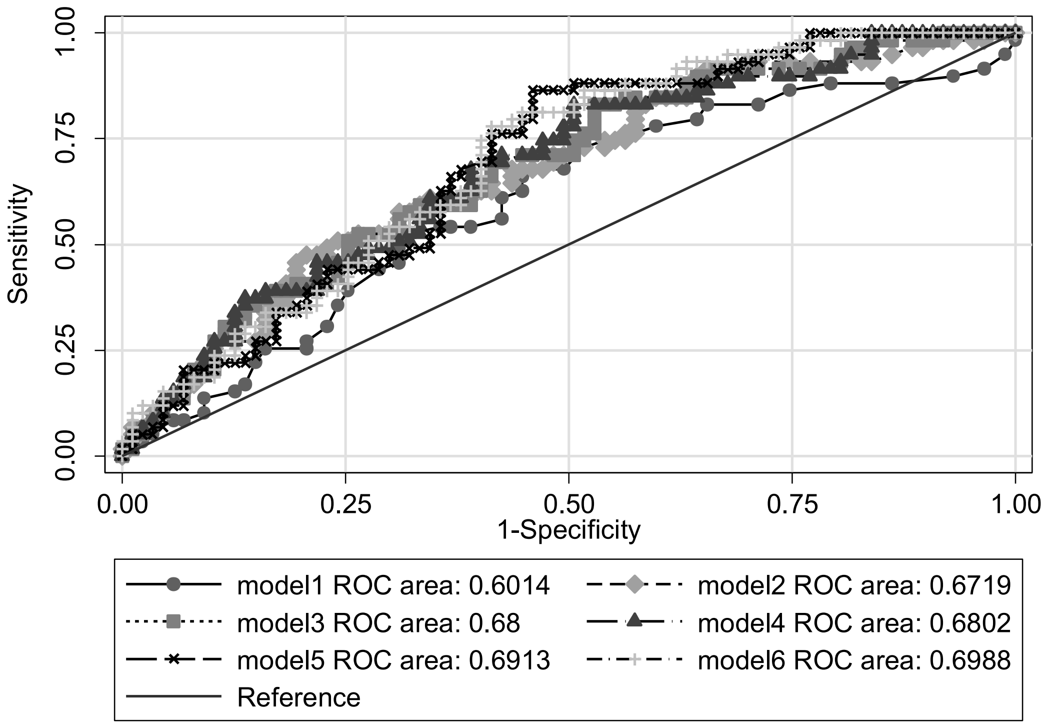

SUPPLEMENTARY MATERIAL III

Comparing the performance of two nested models used in assessing the effect of clinical and lifestyle factors on 2HPP (2-hour postprandial glucose) status.

In investigating the effect of clinical and lifestyle factors, two different nested models were fitted to evaluate the effect of clinical and lifestyle factors on HPP status. Model 11 consists of only clinical and lifestyle factor and model 12 integrates both clinical, lifestyle factor and socio-demographic characteristics. The performance of these two models was evaluated with the Area under Receiver Operating Characteristic Curve (AUROC) and Akaike Information Criterion (AIC). There was statistical significance difference in AUROC between the two nested models ($\chi^2 = 5.60, \mathrm{p} = 0.018$), the best performing model was model 12 (AUROC=73.39%, AIC=216.85).

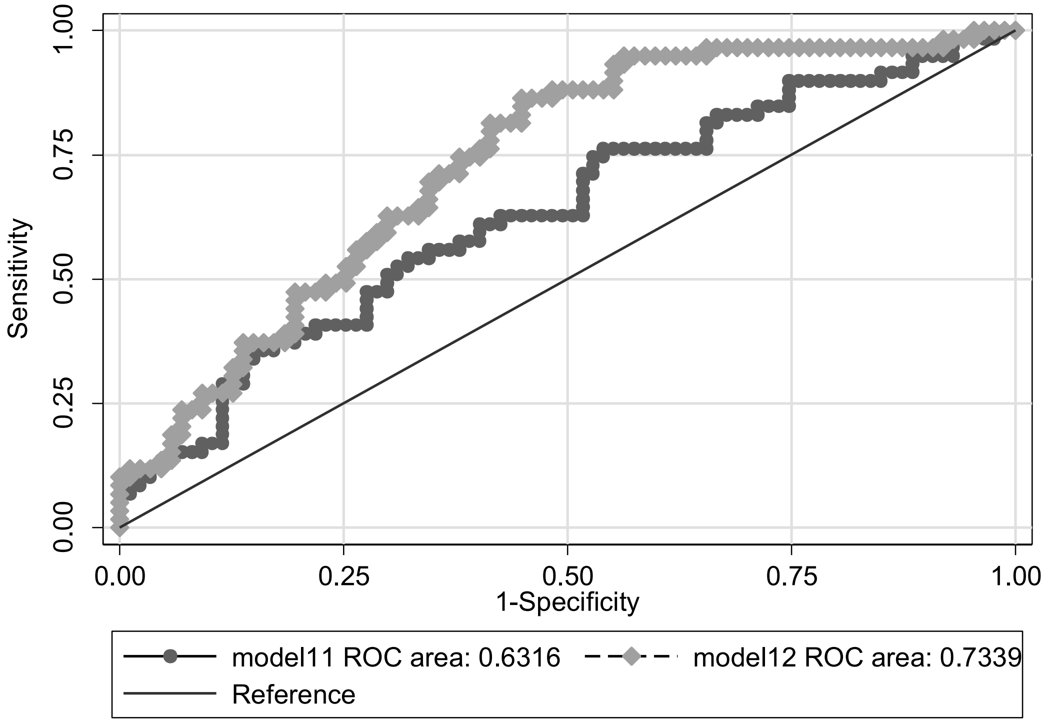

Supplement: Supplementary Materials — I, II: comparison of the performance of six nested models used in assessing the effect of sociodemographic factors on 2HPP (2-hour postprandial glucose) status. III: comparison of the performance of two nested models used in assessing the effect of clinical and lifestyle factors on 2HPP (2-hour postprandial glucose) status. [file 1830372.f1.pdf]
